# Are the functional outcomes really inferior following unicondylar knee arthroplasty in patients with partial-thickness cartilage loss?

**DOI:** 10.5152/j.aott.2021.21093

**Published:** 2021-11-01

**Authors:** Anıl Pulatkan, Fatih Yıldız, Vahdet Uçan, Nurzat Elmalı, İbrahim Tuncay

**Affiliations:** Department of Orthopedics and Traumatology, Bezmialem Foundation University, İstanbul, Turkey

**Keywords:** Unicondylar knee arthroplasty, Medial Oxford knee, Partial thickness cartilage loss, Full thickness cartilage loss, Clinical outcome

## Abstract

**Objective:**

The main indication for medial Unicondylar Knee Arthroplasty (UKA) is Full-Thickness Cartilage Loss (FTCL) in the isolated medial compartment of the knee. However, controversial outcomes were reported in patients with Partial-Thickness Cartilage Loss (PTCL). The aim of this study is to compare PTCL and FTCL based on intraoperative findings in medial UKA in terms of functional outcomes and complication rates requiring reoperation and revision.

**Methods:**

Two hundred and fifteen knees of 174 patients who underwent mobile-bearing UKA between October 2014 and February 2018 for the diagnosis of symptomatic anteromedial osteoarthritis were evaluated retrospectively. A single senior surgeon evaluated the type of cartilage loss in the medial compartment intraoperatively according to the International Cartilage Repair Society classification system. Clinical outcomes were evaluated using Oxford Knee Score (OKS) and International Knee Documentation Committee (IKDC) score pre- and post-operatively at the last follow-up. Patients with PTCL and FTCL were compared in terms of their pre- and post-operative OKS and IKDC scores, and their improvements, as well as complication rates requiring reoperation and revision.

**Results:**

The mean follow-up time was 33.1 ± 5.3 months. The PTCL (n = 80) and FTCL (n = 135) groups were statistically similar in terms of age (*P* = 0.41), gender (*P* = 0.921), body mass index (*P* = 0.165), bilaterality (*P* = 0.111), American Society of Anesthesiologists physical status (*P* = 0.218), Charlson Comorbidity Index (*P* = 0.74), and post-operative follow-up (*P* = 0.167). The mean pre-operative OKS and IKDC scores were improved from 24.5 ± 4.1 and 39.9 ± 5 to 40.3 ± 3.6 and 73.9 ± 7.7 at the last follow-up, respectively (*P* < 0.001). Pre-operative OKS and IKDC scores were superior in favor of the PTCL group. However, no significant difference was found between the groups in terms of post-operative OKS (*P* = 0.53) and IKDC (*P* = 0.975) scores, and their improvements (OKS, *P* = 0.953; IKDC, *P* = 0.536). The complication rates requiring reoperation was 5% (n = 11) in all patients. Of these, 9% (n = 7) from the PTCL group and 3% (n = 4) from the FTCL group were reoperated. Nevertheless, no significant difference was found between the groups (*P* = 0.105).

**Conclusion:**

In PTCL, medial UKA is a reliable surgery in terms of functional outcomes, the same as in FTCL; however, its complication rates requiring reoperation is higher without statistical significance.

**Level of Evidence:**

Level III, Therapeutic Study

## Introduction

Unicondylar Knee Arthroplasty (UKA) is a successful and effective alternative treatment modality in the treatment of symptomatic end-stage Anteromedial Knee Osteoarthritis (AMOA).^1,2^ Despite high complication and revision rates of UKA surgery compared to Total Knee Arthroplasty (TKA) at the previous times, successful results have been achieved with changes in prosthesis design, appropriate patient selection, and surgical technique.^3,4^ Its popularity has been increased in recent years due to its good and excellent long-term results and low rates of morbidity and mortality.^5,6^

The classic indications of UKA surgery were defined by Kozinn and Scott in 1989 as isolated medial compartment Osteoarthritis (OA), aged less than 60 years, weight under 82 kg, low functional capacity, pre-operative flexion range more than 90°, flexion contracture less than 5°, angular deformity less than 15°, intact cruciate ligaments, and minimal pain at rest.^7^ Some surgeons still use these criteria for patient selection; on the other hand, the Oxford group revised the indications for a mobile-bearing prosthesis as AMOA with medial Full-Thickness Cartilage Loss (FTCL), which is defined as bone-on-bone, correctable varus deformity, non-existence of lateral cartilage defect, intact anterior cruciate, and both collateral ligaments.^8-^^11^ Although the designers recommend not to use the Oxford knee system in patients with Partial-Thickness Cartilage Loss (PTCL),^12–14^ some authors state that successful results and high patient satisfaction can be obtained in patients with PTCL of the medial compartment.^15–17^ Therefore, the literature is controversial about the effectiveness of medial UKA in patients with PTCL and still needs to be cleared.

The hypothesis of this study was that the improvements in clinical outcomes in patients with PTCL should be similar to those with FTCL after UKA surgery. The aim of this study was to compare the functional outcomes and complication rates requiring reoperation and revision of the patients with PTCL and FTCL according to the intraoperative findings.

## Materials and Methods

### Study design

This single-center retrospective comparative study was conducted from October 2014 to February 2018 in the Department of Orthopaedics and Traumatology of Bezmialem Vakıf University (İstanbul, Turkey) for UKA of AMOA. Institutional Review Board approval was obtained (No: 45.446.446-010.99-5866). Hospital data, patient records, and surgical reports were reviewed to confirm age, gender, Body Mass Index (BMI), American Society of Anesthesiologists (ASA) physical status classification, Charlson Comorbidity Index (CCI), clinical examination, pre- and post-operative imaging, diagnosis, date of operation, pre- and post-operative functional scores, intraoperative findings, and complications requiring reoperation and revision surgery.^18^ If a patient underwent bilateral UKA application, each joint was considered as a separate case. Informed consent was obtained from all participating patients.

### Patients selection

Patients with primary AMOA, who had no previous knee surgery, underwent mobile-bearing UKA and were followed more than 2 years, were included in the study. Patients who underwent medial UKA due to secondary OA, lateral UKA patients, and patients with a follow-up period of less than 2 years were excluded from the study. The indications for UKA in this study were PTCL or FTCL AMOA, which were unresponsive to at least 6 months of conservative treatment methods, with correctable varus deformity, non-existence of lateral cartilage defect, intact anterior cruciate, and both collateral ligaments.

### Pre-operative clinical and radiological assessment

During the pre-operative period, patients were examined for the localization of pain, medial, and lateral joint line tenderness with palpation, patellofemoral compression test, passive correction of the varus deformity, and Range of Motion (ROM) of the knee.

Radiologic evaluations were done using the standing Anteroposterior (AP) and lateral radiographs. For the standing AP radiographs, the knee was positioned so that the patella was in the middle of the knee. The X-ray tube was placed 100 cm away from the patient and centralized on the inferior pole of the patella. On the lateral radiograph, the wear on the posterior tibia plateau was evaluated, and since posterior cartilage loss suggests Anterior Cruciate Ligament (ACL) deficiency, these patients were not applied UKA. Long Leg Radiographs (LLR) were taken in all patients pre- and post-operatively, in which Mechanical Axis Deviation (MAD) was measured by a radiologist.

### Surgical technique

All of the patients were operated by a single senior surgeon (IT) under general or regional anesthesia with the same surgical technique. Through the medial parapatellar short incision, ACL, lateral and patellofemoral compartment were evaluated and found to be intact. Medial compartment OA was graded according to the International Cartilage Repair Society (ICRS) classification system (Table 1).^19^ ICRS Grade 4 cartilage loss in both distal femur and proximal tibia was determined as FTCL. All cartilage losses outside this condition in the distal femur and proximal tibia were defined as PTCL. The intraoperative indication for UKA surgery was AMOA (ICRS Grade 3 or 4), completely intact cartilage in the lateral compartment, less than Grade 3 OA in the lateral facet of the patella according to the ICRS classification system, and intact ligaments around the joint, including ACL. Femoral and tibial cuts were made with the same technique using “microplasty instrumentation”. Uncemented femoral and tibial components were placed in all patients (Oxford^®^ Phase-3 UKA, Zimmer-Biomet, Warsaw, IN, USA) after Ranawat cocktail injections.^20^ After the capsule was closed, 1 gram of intraarticular tranexamic acid was administered to the joint, and the skin was closed with intracutaneous suture without the use of hemovac drain.

### Follow-up protocol

All patients were allowed full weight-bearing and started ROM training on that day. With ROM training, it was primarily aimed to improve quadriceps muscle strength, increase ROM and enhance the gait of the patients. Each patient was discharged from the hospital within 2 days after the operation with a home rehabilitation program. Routine follow-up was performed at 2 weeks, 6 weeks, 6 months, 1 year, and annually thereafter.

### Functional outcome assessment

Functional outcomes were evaluated using Oxford Knee Score (OKS) and International Knee Documentation Committee (IKDC) score by a research physiotherapist without prior knowledge of the surgical reports and radiological imaging pre- and post-operatively at the last follow-up.^21–24^ The improvements in the functional scores were noted as delta scores. Patients with FTCL and PTCL were compared in terms of pre- and post-operative OKS and IKDC scores, and their improvements, as well as complication rates requiring reoperation and revision. After UKA, all surgical procedures were defined as reoperation, whereas removal of components and application of TKA as revision.

A priori statistical power analysis was performed to calculate the sample size. A difference of between a mean of 1 ± 2 points in the OKS was defined as the minimal clinically relevant difference. With a power of 80%, a 95% confidence level, and an alpha value of 0.05, the results of the power analysis determined a sample size of 64 in each group.

### Statistical analyses

A statistical software package was used to analyze the data (SPSS, Statistical Package for the Social Sciences, version 20.0, IBM, Armonk, NY, USA). Concordance of the continuous data to normal distribution was tested by Shapiro–Wilk test. Two-group comparisons were performed using the Student's *t*-test and Mann–Whitney *U*-test. Categorical comparisons were performed using Fisher’s exact and Chi-square test. In a comparison of the improvements in the functional scores between the groups, the Analysis of Covariance (ANCOVA) was used in which pre-operative values were as the covariate. Continuous variables were expressed with median (minimum-maximum) and mean ± standard deviation values. Categorical variables were expressed with frequency (percentage) values. Results were reported with related *P*-values, and *P*-values less than 0.05 were considered to be statistically significant.

## Results

Hospital data of 354 UKA patients were scanned. Among them patients, who were followed up less than 2 years (n = 64), underwent fixed-bearing UKA (n = 47), had secondary OA, the etiology of which was tibia plateau fracture (n = 3), previous total/subtotal meniscectomy (n = 6), osteonecrosis (n = 15), and without enough documentatiton (n = 45) were excluded. The remaining 215 knees of 174 patients who had at least 2 years of follow-up for the diagnosis of symptomatic AMOA and underwent Oxford^®^ UKA with enough documentation were included. Of the 174 patients, 133 patients underwent unilateral, 21 patients had staged, and 20 patients had single-stage bilateral UKA. The mean age of the patients was 65 ± 4 years (Table 2). According to the gender, 56% (n = 120) of the patients were female, and 44% (n = 95) were male. The mean BMI of patients was 29.4 ± 2.3, and the mean follow-up period was 33.1 ± 5.3 months. The median ASA score of the patients was 1 [range, 1-3], and the average CCI score was 3.6 ± 1.3. According to the intraoperative findings, medial compartment OA was classified as PTCL in 80 knees and FTCL in 135 knees. The mean MAD value in LLR decreased from 20.5 ± 10 mm pre-operatively to 14 ± 6.1 mm post-operatively.

### Functional outcomes

The mean pre-operative OKS and IKDC scores were improved from 24.5 ± 4.1 and 39.9 ± 5 to 40.3 ± 3.6 and 73.9 ± 7.7 at the last follow-up, respectively (*P* < 0.001). There was a statistically significant difference between the PTCL and FTCL groups in terms of pre-operative OKS (*P* = 0.012) and IKDC scores (*P* = 0.017) in favor of the PTCL group. However, no significant difference was found between the groups in terms of post-operative OKS (*P* = 0.53) and IKDC (*P* = 0.975) scores, and their improvements (OKS, *P* = 0.953; IKDC, *P* = 0.536).

### Complications

The rate of complications requiring reoperation was 5% (n = 11) in all patients. Among all patients, 9% (n = 7) of the PTCL group and 3% (n = 4) of the FTCL group were reoperated. Besides, 6% (n = 5) of the PTCL group and 2% (n = 3) of the FTCL group were revised. However, no significant difference was found in the rate of complications requiring reoperation (*P* = 0.105) and revision (*P* = 0.151) between the groups.

In the PTCL group, a total of three patients were reoperated because of insert dislocation; two of them had revision surgery to TKA, and the other one had thicker insert exchange. One patient had arthroscopic debridement because of impingement of insert with femoral condyle while in extension, and a joint body was removed. Two patients underwent revision surgery to TKA because of tibial component loosening with tibial osteolysis, and one patient underwent two-stage revision surgery due to periprosthetic infection.

In the FTCL group, a total of two patients underwent reoperation due to insert dislocation; one patient had a thicker insert exchange and the other one underwent revision surgery to TKA. One patient had undergone two-stage revision arthroplasty because of periprosthetic infection, and another revision to TKA was performed because of loosening of the femoral component with femoral osteolysis (Table 3).

## Discussion

The significant finding of this study is the similarity of post-operative OKS and IKDC functional scores and their improvements in patients with PTCL compared to FTCL in UKA surgery, although there was a significant difference in pre-operative functional scores in favor of the PTCL group. On the other hand, the rate of complications requiring reoperation and revision in the PTCL group was higher than that in the FTCL group without statistical significance.

The literature is controversial in terms of the negative effect of PTCL in medial UKA according to pre-operative imaging or intraoperative findings.^12,14,15,17,25^ In a recent study by Berend et al.,^15^ 92 knees (61%) with FTCL were compared to 60 knees (39%) with PTCL according to Magnetic Resonance Imaging (MRI) and/or radiographs and no difference was found with regard to the Knee, Pain and Function scores of Knee Society, post-operative University of California, Los Angeles (UCLA) activity scores and revision rates. These results are similar to our study as there was no difference between PTCL and FTCL groups in terms of improvements in the functional scores and revision rates. On the other hand, Knifsund et al.^25^ compared the revision rates of UKA in patients with pre-operative Kellgren-Lawrence (K-L) Grade 0-2 (n = 110) and with Grade 3-4 (n = 160) at a single institute, and they stated that the patients with K-L 0-2 had a higher risk for revision. Interestingly, the most common reason for revisions was component loosening, which may not have a direct relation to the type of cartilage loss. In addition, the effect of procedure volume per surgeon on revision rate was not separately assessed.^25^ The main difference and strength of our study is that the type of cartilage loss in the medial compartment was determined according to the intraoperative finding and functional outcomes of the patients were independent of the surgeon. However, Maier et al.^17^ compared clinical outcomes of UKA in patients with PTCL and FTCL according to the pre-operative radiological imaging in 64 matched patients. They found no significant difference between the groups in terms of pre-operative (25 vs 25) and post-operative (41 vs 42) OKS, and in the mean changes of OKS (16 vs 17). They also compared American Knee Society Scores (AKSS), pain on the Visual Analog Scale (VAS), and ROMs between the groups and found no difference in minimum 2 years of follow-up. Five patients with PTCL had a revision, whereas there were only two in the FTCL group. Although the revision rate in the PTCL group was higher, there was no statistically significant difference between the groups (*P* = 0.095). Likewise, in our study, the reoperation rate of patients with PTCL (n = 7, 9%) was found to be higher than that of patients with FTCL (n = 4, 3%) without significant difference (*P* = 0.105). It is possible to figure out that the studies with more patients in this low revision rate surgery will bring more clarity to the comparison of complication rates between FTCL and PTCL.

In another study with similar results to our study, Carlson et al.^16^ compared the clinical outcomes and survivorship of mobile-bearing UKA in patients with bone-on-bone contact and patients without bone-on-bone contact. There was no significant difference in functional scores (*P* = 0.9) and revision rates (*P* = 0.42) between the groups. There were five revisions in the group with bone-on-bone contact and two revisions in the group without bone-on-bone contact. They concluded that patients with bone-on-bone contact had similar outcomes of mobile-bearing UKA compared with patients without bone-on-bone contact.

In contrary to similar functional outcomes of PTCL and FTCL in UKA, there are some studies in the literature showing the superiority of FTCL with regard to post-operative functional scores. Pandit et al.^12^ compared 29 Oxford UKA patients with PTCL in the medial compartment with the same number of matched patients who had Bone Exposed (BE) and Bone Loss (BL) and published an average of 2 years of follow-up results. They stated that change in OKS was significantly lower in patients with PTCL (36 ± 10) than that in patients with FTCL of both BE (43 ± 4) and BL (43 ± 5) groups (*P* < 0.001). In addition, they recommended that Oxford UKA should be used only if there is FTCL in the medial compartment. Similarly, Hamilton et al. compared the medium-term results of 94 consecutive UKAs with PTCL to FTCL.^14^ They stated that patients with PTCL who had poor results were younger and pre-operative functional scores were inferior. At 5 years, the rate of reoperation in knees with PTCL was 10.9%, three times that of those undertaken in knees with FTCL on both surfaces, 3.9%. In these studies, matched patient groups with similar demographic characteristics and their similar pre-operative functional scores were used. Thus, it may cause a bias at the patient selection in two different groups with different pre-operative patient scores and may not accurately reflect the comparison between the selected group of patients and the actual group population. In our study, both of the pre-operative OKS (*P* = 0.012) and IKDC (*P* = 0.017) scores were superior in favor of the patients with PTCL. In a comparison of the improvements of functional scores, pre-operative values were as a covariate, and there was no statistically significant difference between groups (OKS, *P* = 0.953; IKDC, *P* = 0.536).

This study has several limitations, including its retrospective nature and a small number of patients with a short-term follow-up. Another limitation of the study is that the number of patients with PTCL and FTCL was similar in the study, and the results of this condition do not reflect the outcome of classical UKA indication. In terms of the type of cartilage loss, not considering femur and tibia separately can also be considered a limitation. The absence of interobserver reliability on deciding the type of cartilage loss according to the intraoperative findings may be another limitation. In addition, the focus was not including pre-operative imaging for deciding the type of cartilage loss. ICRS Grade 3 subgroup analysis could not be performed without arthroscopy instruments due to difficulties in the assessment of cartilage status by direct visual examination.

Medial UKA is a reliable treatment method with similar functional outcomes for PTCL (ICRS Grade 3) and FTCL (ICRS Grade 4) in the isolated AMOA. On the other hand, the rate of complications requiring reoperation and revision was higher in patients with PTCL than that in patients with FTCL without statistical significance. Prospective comparative studies involving more patients with longer follow-up will help to determine whether the clinical outcomes and revision rate following UKA are better in patients with FTCL compared to PTCL.

**HIGHLIGHTS**

- The results of unicondylar knee arthroplasty for patients with partial or full thickness cartilage loss are similar.
- Lack of bone-on-bone contact should not be considered a contraindication for unicondylar knee arthroplasty.
- Unicondylar knee arthroplasty is a reliable surgery in patients with partial and full thickness cartilage loss in anteromedial osteoarthritis.

## Figures and Tables

**Table 1.** Modified International Cartilage Repair Society (ICRS) Classification System

Grade	Modified ICRS classification system
**0**	Normal cartilage
**I**	Superficial lesions *A) Soft indentation* *B) And/or superficial fissures and cracks*
**II**	Abnormal lesions extending down to < 50% of cartilage depth
**III**	Severe lesions *A) Cartilage defects extending down > 50% of cartilage depth* *B) As well as down to calcified layer* *C) And down to but not through the subchondral bone* *D) Blisters*
**IV**	Penetration through subchondral bone

**Table 2.** Comparison of patient demographics and outcomes between the groups

	Group PTCL (n = 80)	Group FTCL (n = 135)		
	Descriptive Statistics		*P*	
Age (Years)	64 ± 4	65 ± 4	0.41^a^	
Gender (Female/Male)	45/35	75/60	0.921^b^	
BMI (kg/m^2^)	29.2 ± 2.1	29.6 ± 2.4	0.165^a^	
Bilateral UKA rate (%)	36/80 (45%)	46/135 (34%)	0.111^b^	
Follow-up (Months)	33.7 ± 5.4	32.6 ± 5.2	0.167^a^	
ASA Score	1 [1-3]	1 [1-3]	0.218^c^	
CCI	3.5 ± 1.4	3.6 ± 1.3	0.74^c^	
Preoperative MAD (mm)	19.2 ± 9.5	21.3 ± 10.2	**0.046** ^c^	
Post-operative MAD (mm)	13.1 ± 6.3	14.5 ± 5.9	0.056^c^	
Preoperative IKDC	40.9 ± 5.2	39.2 ± 4.8	**0.017** ^a^	
Post-operative IKDC	73.8 ± 8.4	73.9 ± 7.3	0.975^a^	
ΔIKDC	32.9 ± 8.4	34.6 ± 7.9	0.536^d^	
Preoperative OKS	25.4 ± 3.9	23.9 ± 4.1	**0.012** ^a^	
Post-operative OKS	40.5 ± 3.7	40.2 ± 3.5	0.53^a^	
ΔOKS	15.2 ± 4.8	16.3 ± 4.7	0.953^d^	
Reoperation rate (%)	7/80 (9%)	4/135 (3%)	0.105^e^	
Revision rate (%)	5/80 (6%)	3/135 (2%)	0.151^e^	

ASA, American Society of Anesthesiologists; BMI, Body Mass Index; CCI, Charlson Comorbidity Index; FTCL, Full Thickness Cartilage Loss; IKDC, International Knee Documentation Committee; MAD, Mechanical Axis Deviation; OKS, Oxford Knee Score; PTCL, Partial Thickness Cartilage Loss; UKA, Unicondylar Knee Arthroplasty.

a *Student's t*-test.

b Chi-square test.

c Mann–Whitney *U*-test.

d Analysis of covariance (ANCOVA).

e Fisher’s Exact test.

**Table 3.** Comparison of groups in terms of reoperation details

Complications	UKA (n)
**Group PTCL**	**7**
*Insert dislocation*	*3*
*Tibial component loosening*	*2*
*Prosthetic joint infection*	*1*
*Impingement*	*1*
**Group FTCL**	**4**
*Insert dislocation*	*2*
*Femoral component loosening*	*1*
*Prosthetic joint infection*	*1*

FTCL, Full Thickness Cartilage Loss; PTCL, Partial Thickness Cartilage Loss; UKA, Unicondylar Knee Arthroplasty.
